# A framework for genomic biomarker actionability and its use in clinical decision making

**DOI:** 10.18632/oncoscience.90

**Published:** 2014-10-22

**Authors:** Smruti J. Vidwans, Michelle L. Turski, Filip Janku, Ignacio Garrido-Laguna, Javier Munoz, Richard Schwab, Vivek Subbiah, Jordi Rodon, Razelle Kurzrock

**Affiliations:** ^1^ CollabRx Inc., San Francisco, California, USA; ^2^ Department of Investigational Cancer Therapeutics – a Phase I Clinical Trials Program, The University of Texas MD Anderson Cancer Center Houston, Texas, USA; ^3^ Huntsman Cancer Institute, University of Utah, Salt Lake City, Utah, USA; ^4^ Banner MD Anderson Cancer Center, Gilbert, Arizona, USA; ^5^ Center for Personalized Cancer Therapy, Moores Cancer Center, University of California, San Diego, California, USA; ^6^ Vall d'Hebron Institut d'Oncologia and Universitat Autonoma of Barcelona, Barcelona, Spain

**Keywords:** Biomarker, Clinical decision making, Targeted therapies, Predictive biomarkers

## Abstract

The increasing scope and availability of genetic testing options for patients suffering from cancer has raised questions about how to use results of molecular diagnostics to inform patient care. For some biomarkers (e.g*. BRAF* mutations in melanoma), standards exist that outline treatments for individuals harboring aberrations in the biomarker; however for the vast majority of genomic abnormalities, few guidelines exist. Clinical decision making and the therapeutic approach for a patient with a given cancer characterized by aberrations in different genes may be aided by the use of a biomarker actionability framework that provides levels of evidence regarding whether and how a molecular abnormality can be considered a therapeutically relevant biomarker. A gene may be considered theoretically actionable if it has a basis of actionability, such that clinically available drugs can target a gene product that drives the cancer or is differentially expressed in tumor versus normal elements. Herein, we discuss a possible framework for developing guidelines for actionability, as they relate to genomically-based cancer therapeutics.

## INTRODUCTION

Over the last decade, there has been a breathtaking fall in the cost of genomics, accompanied by a precipitous rise in the efficiency of the technology that enables rapid sequencing of human genomes. As a result, biomarkers, especially those representing genomic alterations, are becoming increasingly vital to the classification and treatment of cancer. Biomarkers are herein defined as measurable molecular or cellular elements linked to a health outcome or state; they can be functionally important in tumors (“oncogenic drivers”) or may be differentially expressed in the malignant versus normal tissue without functional impact (“passengers”).

A biomarker is actionable if it is oncogenic and/or differentially expressed on tumor cells, and a treatment approach can be crafted that mitigates its oncogenic potential and/or permits the recognition and destruction of tumor cells. Driver aberrations can be targeted by interfering with their function, or by exploiting them to identify and select tumor cells for destruction. Passenger biomarkers, however, can also be targeted, if they are used to select cancer cells for destruction by virtue of the targeting agent recognizing differences between cancer and normal elements.

Diagnostic laboratories around the world are now offering tests that sequence either full exomes or gene panels in cancer samples. It is expected that, in the near future, more clinical-grade transcriptomic and proteomic tests will also become available. While genomics have traditionally been performed for research purposes, in the United States, tests performed under the auspices of the Clinical Laboratory Improvement Amendments (CLIA) can be utilized to inform patient care; currently, a variety of genomic tests, from single gene appraisal to multi-panel tests on anywhere from about 10 to over 400 genes [1], full exomic sequencing [2], and transcriptomics [3] are obtainable from CLIA-certified laboratories. The hope is that patients can be matched to approved targeted therapies or clinical trials with investigational targeted therapies on the basis of the molecular profiles of their cancer. However, our understanding of the way in which biomarkers defined by genomics are predictive of therapeutic response lags behind the widespread availability of next generation sequencing (NGS) and other diagnostic tests and the pace of development of targeted therapies.

From the clinical management viewpoint, biomarkers may broadly be divided into three groups. In the first category are a handful of biomarkers that are clinically validated, may be in widely accepted treatment guidelines (such as the NCCN [4] or FDA guidelines [5]) and may have approved drugs that target them. These include *BRAF* V600 mutation for melanoma [6], *EGFR* mutation [7] and *ALK* fusion for lung cancer [8], *HER2* amplification or overexpression for breast cancer [9], and *KRAS* mutation for colorectal cancer [10]. In the second category are biomarkers that have a basis for “actionability” having one or more of the following factors: (i) clinical evidence suggesting responsiveness to one or more drugs or classes when the biomarkers are present; (ii) the biomarkers are direct targets of approved/investigational drugs; (iii) the biomarkers are part of a pathway that can be targeted by approved/investigational drugs; or (iv) the biomarkers bear similarity to other biomarkers that are deemed actionable. Finally, the third category includes biomarkers for which there is no discernable link to actionability.

The framework also includes a rationale for actionability in which strength of evidence for a biomarker is mapped to *highest* strength of evidence for a given cancer. In this paper, we propose an actionability framework and provide our perspective on its use in development of potential treatment approaches as well as clinical research strategies for diverse malignancies.

### Basis of Actionability

A conceptual framework for basis of actionability is outlined in Table 1. Briefly, the framework divides biomarkers into those that do or do not have a function directly important to tumor pathogenesis. It should be kept in mind, however, that these dichotomies are sometimes blurred. For instance, passenger mutations can at times be indirectly related to or responsible for development of functional changes and, regardless, can generate therapeutic vulnerabilities in cancer [11]. Further stratification includes those biomarkers that can be targeted directly versus indirectly by approved versus investigational drugs, and finally those that may be homologous to each of the types of biomarkers mentioned above.

**Table 1:** Basis of Actionability

Biomarker Criteria	Definition of Biomarker Criteria	Example*
Functional in driving the malignancy and can be targeted by approved drug(s)	Biomarker is a direct target of one or more approved drugs, and targeting it will interfere with malignant cell growth.	ALK
Functional in driving the malignancy and can be targeted by investigational drug(s)	Biomarker is a direct target of one or more investigational drugs, and targeting it will interfere with malignant cell growth.	AKT1
Direct component of an actionable pathway that can be targeted by approved and/or investigational drugs	Biomarker may not be directly targeted by approved or investigational drugs, but instead is part of a pathway that drives the malignancy and can be directly targeted by drugs.	PTEN
Indirect component of an actionable pathway that can be targeted by approved and/or investigational drugs	Biomarker itself may not be directly targeted by approved or investigational drugs, but influences the activity or expression of other proteins that can be targeted by either approved and/or investigational drugs.	FBXW7
Homologous to an actionable biomarker that can be either directly or indirectly targeted by approved and/or investigational drugs	Biomarker itself may not be a target for clinically available drugs, but may be homologous to biomarkers that are targetable.	GNAO1
Can be targeted by drug(s) even if the biomarker is not itself functional in driving the malignancy	Biomarker may not be functionally important in the malignancy, yet can be expressed aberrantly or differentially in cancer cells and, hence, exploited for targeted delivery.	CD20, CD30

* See text for more information

#### Biomarker that is functional in driving the malignancy and can be targeted by approved drug(s)

A biomarker may be considered actionable if it is a direct target of one or more approved drugs, and if targeting it will interfere with malignant cell growth. The ALK-inhibitor crizotinib is approved for the treatment of *ALK*-rearranged non-small cell lung cancer [12-14]. It also inhibits MET [15] and ROS1 [16] kinases and may be a potential therapeutic option for cancers with driver aberrations in these genes.

#### Biomarker that is functional in driving the malignancy and can be targeted by investigational drug(s)

A biomarker may be considered actionable if it is a direct target of one or more investigational drugs, and targeting it will interfere with malignant cell growth. For example, several investigational drugs, such as MK-2206 and GDC-0068, inhibit AKT1 [17-19] and clinical trials with these agents may be treatment options to consider for cancers with activating *AKT1* aberrations.

#### Biomarker is a direct component of an actionable pathway that can be targeted by approved and/or investigational drugs

A biomarker may not be directly targeted by approved or investigational drugs, but instead it may be part of a pathway that drives the malignancy and can be directly targeted by drugs. For example, the tumor suppressor PTEN is not currently the target of a drug or class of drugs. However, reduced PTEN function leads to increased activity of the PI3K/AKT/mToR pathway [20-22] that is targeted by drugs from several drug classes [19,23,24].

#### Biomarker is an indirect component of an actionable pathway that can be targeted by approved and/or investigational drugs

A biomarker itself may not be a target for clinically available drugs, but influences the activity or expression of other proteins that can be targeted by either approved and/or investigational drugs. For example, FBXW7 is not a target of clinically available drugs nor is it part of a pathway, but it does regulate the expression of many different proteins because it functions as an ubiquitin ligase that targets proteins for degradation. FBXW7 directly regulates proteins such as mTOR and NOTCH1, both of which are targets of clinically available drugs. Studies have shown that cells deficient for *FBWX7* demonstrate increased levels of active mTOR [25] and NOTCH1 [26,27]. Thus, mTOR or NOTCH1 inhibitors could be effective in ameliorating the consequences of *FBXW7* inactivation in cancer cells.

#### Biomarker is homologous to an actionable biomarker that can be either directly or indirectly targeted by approved or investigational drugs

A biomarker itself may not be a target for clinically available drugs, but may be homologous to biomarkers that are targetable. For example, *GNAO1* is a member of the G-alpha protein family of GTPases and shares homology with *GNAQ* and *GNA11*, biomarkers that predict responsiveness to MEK inhibitors [28,29]. Knowledge about *GNAQ* or *GNA11* may help inform potential treatment approaches for *GNAO1*-aberrant cancers. The other aspect of this homology is the finding of new mutations. For instance, if a new, previously un-described mutation is found in *BRAF*, can that be considered actionable in a manner similar to known *BRAF* mutations? One solution may be to model the homologous biomarker or the new mutation *in silico* and/or perform pre-clinical experiments and determine if it is predicted to be activating in a manner similar to *BRAF* V600E (in the case of a novel *BRAF* mutation) or the homologous biomarker.

#### The presence of the biomarker/aberration can be targeted by drug(s) even if the biomarker is not itself functional in driving the malignancy

A biomarker may not be functionally important in the malignancy, yet can be expressed aberrantly or differentially in cancer cells and, hence, can be exploited for targeted delivery. One example, is the CD20 antigen, a B-cell–specific differentiation antigen expressed on mature B-cells and in most B-cell non-Hodgkin's lymphomas [30]. Several antibodies targeting CD20 exert their anti-tumor effect by complement-dependent cytotoxicity, antibody-dependent cell-mediated cytotoxicity, and/or through antibody binding to antigen leading to anti-proliferative or apoptotic effects on cells expressing the target antigen [30]. Rituximab is a CD20-directed cytolytic antibody approved for CD20-positive Non-Hodgkin's lymphoma or chronic lymphocytic leukemia [31,32]. Another example is the CD30 antigen, which is expressed by normal activated lymphocytes and is highly expressed by some malignant cells [33]. Brentuximab vedotin is an antibody-drug conjugate that consists of an antibody targeting the CD30 antigen linked to a chemotherapeutic agent monomethyl auristatin E (MMAE), which inhibits microtubule polymerization [34]. Brentuximab vedotin is approved for Hodgkin's lymphoma and systemic anaplastic large cell lymphoma, both of which express high levels of CD30 [14].

### Rationale for Actionability

The possible rationales for actionability are summarized in Table 2. Briefly, rationales range from a drug approved with a companion diagnostic, through NCCN guidelines for a biomarker, to clinical trials with a biomarker, pre-clinical evidence for a biomarker, and evidence in hereditary (genetic) disease regarding a biomarker with possible extrapolation to malignancy. Biomarkers approved as companion diagnostics or those in FDA and NCCN guidelines have the highest level of support for their actionability.

**Table 2:** Rational for Actionability

Biomarker Criteria	Definition of Biomarker Criteria	Example*
Drug approved with companion diagnostic	A drug is approved for cancers with an aberration in that biomarker.	BRAF, HER2, KIT
Therapeutic approach outlined in treatment guidelines (e.g. NCCN guidelines)	Standard clinical treatment guidelines recommend that cancers with aberrations in that biomarker should (or should not) be treated with certain drugs and drug classes.	KRAS, NRAS,EGFR
Clinical evidence indicating responsiveness to drug class(es)	Available clinical data suggests that aberrations within the biomarker may be predictive of therapeutic response.	BRAF, PIK3CA, HER2, TP53
Clinical trials with biomarker aberration as an inclusion criteria	Clinical trials seek to enroll patients whose cancers harbor specific aberrations in that biomarker.	CDK6
Pre-clinical evidence indicating responsiveness to drug class(es)	Available pre-clinical data suggests that aberrations within the biomarker may be predictive of therapeutic response.	MAP3K9
Evidence in genetic disease with biomarker aberration	Available clinical data on the therapeutic response of the biomarker within the context of a non-cancer disease.	TSC1
No evidence	A biomarker is not considered to be actionable if there is no data on the above mentioned criteria for that biomarker.	ADAMTS20

* See text for more information

#### Drug approved with companion diagnostic

Several drugs are now approved for cancers with specific aberrations thus making the corresponding biomarkers actionable. For example, vemurafenib [14] and dabrafenib [35] are approved for the treatment of melanoma with *BRAF* V600E aberrations; trametinib is approved for melanoma with *BRAF* V600E or *BRAF* V600K aberrations [36]. Trastuzumab, pertuzumab, lapatinib, and trastuzumab emtansine are approved for the treatment of breast cancer with HER2 overexpression [37], and imatinib is approved for the treatment of KIT-positive gastrointestinal stromal tumors (GISTs) [38].

#### Therapeutic approach outlined in treatment guidelines (such as FDA or NCCN guidelines)

A biomarker may be considered actionable if standard clinical treatment guidelines recommend that cancers with aberrations in that biomarker should (or should not) be treated with certain drugs and drug classes. For example, standard treatment guidelines recommend *KRAS* and *NRAS* testing for colorectal cancer patients and that patients with *KRAS-* and *NRAS-*mutated colorectal cancer not be treated with epidermal growth factor receptor inhibitors such as cetuximab and panitumumab [39-44].

Another example is EGFR, where standard treatment guidelines recommend testing for EGFR mutations in patients with non-small cell lung cancer. Patients whose tumors harbor sensitizing EGFR mutations should receive the EGFR tyrosine kinase inhibitors erlotinib, gefitinib, or afatinib as first-line therapy [45-50].

#### Clinical evidence indicating responsiveness to drug class(es)

A biomarker may be considered actionable if available clinical data suggests that the biomarker is predictive of therapeutic response. Clinical data may come from different sources, with phase 3 studies providing the most robust evidence, and phase 1 or 2 studies or retrospective studies or registry data providing less definitive evidence. Importantly, genomics has unveiled substantial complexity and heterogeneity associated with cancers. As such, more “rare” tumor subtypes are being recognized and it is increasingly evident that the ability to perform randomized trials to ascertain efficacy for these patients is a monumental hurdle. This has been acknowledged by regulatory agencies as well. For instance, the FDA approved the multikinase inhibitor imatinib in ultra-rare disorders such aggressive systemic mastocytosis, dermatofibrosarcoma protuberans, PDGFR-rearranged myeloprofierative disease and others based on high response rates in small numbers of patients bearing the cognate biomarker that participated in phase 2 studies or were described in case reports [51]. Balancing the need for validated efficacy data versus the reality that cancers may be increasingly stratified by their biomarkers into rare subsets is therefore a defining issue for cancer therapeutics. Examples of the type of clinical evidence that might be collected follows:

a. Phase 3 studies. For instance, in a phase 3 clinical trial, melanoma patients with *BRAF* V600E-mutated melanoma had higher clinical response rates to treatment with the BRAF inhibitor, vemurafenib, than the chemotherapy, dacarbazine [6].
b. Phase 1 or 2 studies. For example, in phase 1 clinical trials, patients with *PIK3CA*-aberrant cancer had a higher clinical response rate to treatment with PI3K/AKT/mTOR inhibitors than patients who lacked these aberrations [52,53].
c. Case reports. For example, a non-small cell lung cancer patient whose tumor harbored a *HER2* exon 20 mutation showed tumor shrinkage on a treatment regimen that included anti-HER2 drugs [54,55]. Another example is a patient diagnosed with spindle cell neoplasm harboring a *KIAA1549-BRAF* fusion protein and *PTEN* deletion that responded to a RAF and mTOR kinase targeted combination inhibitor therapy [56].
d. Retrospective studies. For example, a retrospective study reported that patients with advanced cancers harboring *TP53* aberrations experienced longer progression-free survival on treatment regimens containing bevacizumab [57].
e. Registry data. For example, genomic data has been compiled through The Cancer Genome Atlas (TCGA). This database provides a comprehensive overview of the genomic aberrations in a wide variety of cancers [58]. Similarly, registry of clinical observations could be collated. For instance, a breast cancer registry pilot program funded by Susan G. Komen for the Cure was compiled from September 2009 to December 2010, based on twenty diverse oncology practices. A major goal was to generate an anonymized breast cancer registry database to inform future quality of care and research initiatives. The American Society of Clinical Oncology (ASCO) has also envisioned creating observational registries to inform patient care, including data on panomics and precision medicine-based therapies and outcomes [59-61].
f. Navigation or umbrella studies (which may be histology-agnostic), that demonstrate that patients with a specific biomarker may respond to certain treatments [62].

#### Clinical trials with biomarker aberration as an inclusion criteria

An increasing number of clinical trials seek to enroll patients whose cancers harbor specific aberrations. For example, the trial NCT01164995 (Study With Wee-1 inhibitor MK-1775 and Carboplatin to Treat p53 Mutated Refractory and Resistant Ovarian Cancer) is seeking ovarian cancer patients whose tumors harbor mutations in *TP53*. The rationale for this study is that pre-clinical data suggests that abrogation of the G2 checkpoint by inhibition of Wee-1 kinase results in sensitization of p53-deficient tumor cells to DNA-damaging agents [63]. *TP53* may be considered actionable because patients with *TP53*-aberrant ovarian cancer could enroll in such clinical trials. Other trials seeking to match aberrations in specific biomarkers with therapies that either directly target the biomarker or indirectly with drugs that target downstream effects of the aberrant biomarker include the National Cancer Institute's NCI-MPACT (NCT01827384) and LUNG-MAP (NCT02154490) trials. NCI-MPACT seeks to enroll solid tumor patients with mutations or amplifications in specific pathways, with PARP inhibitor ABT-888 or MK-1175 plus carboplatin given to patients with tumors harboring defects in the DNA repair pathway, the mTOR inhibitor everolimus given to patients with tumors having alterations in the PI3K pathway, and MEK inhibitor trametinib given to patients with tumors that have alterations in the RAS/RAF/MEK pathway. The LUNG-MAP trial is specifically for squamous cell lung cancer patients that is testing 4 different targeted therapies and an anti-PD-L1 therapy for patients whose tumors harbor alterations in a number of different biomarkers including *PIK3CA*, *CDK4*, *CDK6*, *CCND1*, *CCND2*, *CCND3*, *FGFR1*, *FGFR2*, *FGFR3*, and *HGF/c-MET*.

#### Pre-clinical evidence indicating responsiveness to drug class(es)

A biomarker may be considered actionable based on pre-clinical data indicating that an aberration or class of aberrations (e.g activating or inactivating) in the biomarker responds to a specific drug or drug class. For example, viability of lung cancer cells with an activating *MAP3K9* aberration was inhibited by treatment with a MEK inhibitor [64].

#### Evidence in genetic disease with biomarker aberration

A biomarker may also be considered actionable if there is clinical data on the therapeutic response of the biomarker within the context of a non-cancer disease. For example, inactivating mutations in the gene *TSC1* result in upregulation of mTOR and cause the disease Tuberous Sclerosis Complex [65]. The drug everolimus, an mTOR inhibitor, is approved for the treatment of tuberous sclerosis [66]. Inactivating mutations in *TSC1* are also common in cancer [67] and based on the clinical evidence in a related genetic disease, mTOR inhibitors might also be considered as a treatment option.

### Use of Actionability Framework in Clinical Decision-Making

Interpretation and use of molecular data in clinical decision-making often involves extrapolating predictive data from the tumor site of origin with the highest strength of evidence to a different histology under consideration by the physician. This may be complicated by several issues described below.

#### Conflicting data in several cancers

*BRAF* V600E mutation are predictive of response to BRAF inhibitors in melanoma and have been reported to predict response in other *BRAF*-positive malignancies such as hairy cell leukemia [68], histiocytosis, [69] and thyroid cancer [70,71], but not in colorectal cancer [72]. Is the lack of response in colorectal cancer due to the presence of additional anomalies that co-occur with *BRAF* mutations, or because BRAF inhibition causes feedback activation of EGFR [72], or because *BRAF* mutations are not driver abnormalities in colorectal cancer? Given these data, should patients with another cancer harboring *BRAF* mutations be treated with BRAF inhibitors? What are the considerations that might inform this decision?

One consideration is increasing evidence that molecular aberrations often do not segregate by histetology; for instance, *BRAF* mutations can be found in a subset of patients with almost any cancer [73]. Indeed, it may be near impossible to perform definitive clinical trials of BRAF inhibitors in each histology that harbors *BRAF* mutations. Historically, when approving or accepting a drug for therapeutic application in a specific tumor type as defined by histology, the drug is not expected to be effective for all patients with that tumor type; indeed 80% or more of patients may not respond/benefit. Therefore, if we define “tumor type” by the presence of a biomarker (rather than by organ of origin), the same principles may apply. The main question that would need to be ascertained might be whether or not patients who have that tumor type (e.g. *BRAF*-mutated cancer) benefit from the therapy overall, rather than if there are salutary effects for each histologic subtype or patient. As with use of drugs when tumor type is classified histologically, an important consideration for therapy in tumor types classified on the basis of a biomarker might be comparison of potential efficacy to other treatment options available to the patient.

#### Aberrations of unknown significance

What is the significance/relevance of alternative aberrations in a validated biomarker like *BRAF* whose impact is not known? For instance, if a new, previously un-described mutation is found in *BRAF*, can that be considered actionable in a manner similar to *BRAF* V600E mutations. One solution may be to model the aberration *in silico* to determine if it is predicted to be activating in a manner similar to *BRAF* V600E or characterize the functional effects of the aberration using *in-vitro* or *in-*vivo experiments. However, validation of such approaches are needed.

#### Tumors are a complex collection of genetic alterations

A recent study examined the distributions of mutation frequencies, types and contexts across many different cancer histologies using a panel of 127 significantly mutated genes from well known (e.g. receptor tyrosine kinase signaling pathways) or emerging (e.g. histone modification) cellular processes in cancer. The study reported that most tumors have anywhere from 2 to 6 aberrations in the studied genes [73]. The complexity is further amplified when testing for non-mutation based aberrations in a patient's tumor. For example, in a recent single patient case study of metastatic malignant phyllodes tumor, a comprehensive molecular analysis was performed by using multiple Clinical Laboratory Improvement Amendments (CLIA)-certified labs on the same tumor sample including next-generation sequencing, whole-genome array-based comparative genomic hybridization, proteomics, and immunohistochemistry, which revealed mutations (missense and nonsense), gene amplifications, gene deletions, and aberrant expression patterns in 13 different genes [74]. Given this, how does an oncologist formulate a treatment approach or a clinical researcher design a research strategy that comprehensively addresses all the aberrations detected in a patient's tumor specimen? One potential solution is a systems biology approach to examine the phenotypic convergence of a collection of aberrations in a tumor at the molecular level, i.e. are one or more pathways unregulated, and to determine a treatment approach or research strategy based on identifying oncogenic hubs. In many instances even in patients who are exceptional responders to a therapy, a genomic basis of drug sensitivity may not be identified and in such cases proteomic or transcriptomic approaches may be helpful in identifying pathways of resistance and / or response [75,76].

## CONCLUSIONS

Diagnostic laboratories are now offering tests that interrogate tumors by sequencing either gene panels or full exomes/genomes in cancer samples. The degree to which genomic biomarkers can be successfully prosecuted is, however, not yet fully elucidated. For some of these tests, like *BRAF* V600E, it is clear that the aberration is a functionally important mutation, approved drugs are available to target it, and specific guidelines for the use of the molecular diagnostic and the cognate drug are available. However, for the majority of these tests, the extent to which the test data are usable is less clear. To optimize deployment of molecular diagnostic profiling, more research is needed. Clinical trials or registries designed to examine scientifically informed theories as to the predictive value of certain biomarkers for carefully selected targeted therapies or to catalogue the genetic constituency of tumors and the response to various treatments are ways in which the clinical value of “new” biomarkers can be optimized, and test information about a biomarker can be exploited to inform patient care. We have provided a perspective on actionability of biomarkers that could be used to highlight areas of needed pre-clinical, clinical, or pharmaceutical research, as well as to inform discussions regarding consensus guidelines in this new frontier of biomarker discovery and utilization.
